# Changes in quality of life and its associated factors among illicit drug users in Vietnamese mountainous provinces: a 12-month follow-up study

**DOI:** 10.1186/s13011-020-00265-7

**Published:** 2020-03-18

**Authors:** Bach Xuan Tran, Mackenzie Moir, Tam Minh Thi Nguyen, Ha Ngoc Do, Giang Thu Vu, Anh Kim Dang, Giang Hai Ha, Trang Huyen Thi Nguyen, Hoang Quan Vuong, Tung Manh Ho, Nhue Van Dam, Trang Thu Vuong, Carl A. Latkin, Cyrus S. H. Ho, Roger C. M. Ho

**Affiliations:** 1 Institute for Preventive Medicine and Public Health, Hanoi Medical University, No. 1 Ton That Tung Street, Hanoi, 100000 Vietnam; 2 Bloomberg School of Public Health, Johns Hopkins University, Baltimore, MD 21205 USA; 3 School of Public Health, University of Alberta, Edmonton, Alberta T6G 1C9 Canada; 4 Vietnam Authority of HIV/AIDS Control, Ministry of Health, Hanoi, 100000 Vietnam; 5 Youth Research Institute, Ho Chi Minh Communist Youth Union, Hanoi, 100000 Vietnam; 6 Center of Excellence in Evidence-based Medicine, Nguyen Tat Thanh University, Ho Chi Minh City, 700000 Vietnam; 7 Institute for Global Health Innovations, Duy Tan University, Da Nang, 550000 Vietnam; 8 Center of Excellence in Pharmacoeconomics and Management, Nguyen Tat Thanh University, Ho Chi Minh City, 700000 Vietnam; 9 Centre for Interdisciplinary Social Research, Phenikaa University, Yen Nghia, Ha Dong, Hanoi, 100000 Vietnam; 10 National Economics University, Hanoi, 100000 Vietnam; 11 Sciences Po Paris, Campus de Dijon, 21000 Dijon, France; 12 Department of Psychological Medicine, National University Hospital, Singapore, 119074 Singapore; 13 Center of Excellence in Behavioral Medicine, Nguyen Tat Thanh University, Ho Chi Minh City, 700000 Vietnam; 14 Department of Psychological Medicine, Yong Loo Lin School of Medicine, National University of Singapore, Singapore, 119228 Singapore; 15 Institute for Health Innovation and Technology (iHealthtech), National University of Singapore, Singapore, 119077 Singapore

**Keywords:** Vietnam, Methadone maintenance therapy, Drug abuse, Injection drug use, Health-related quality of life, WHOQoL-BREF

## Abstract

**Background:**

Sustainability of methadone maintenance treatment (MMT) program involves regularly assessing its impact on health and social outcomes of drug users in different settings, particularly in disadvantaged areas. In this study, we evaluated the change in quality of life (QoL), and identified associated factors amongst drug users over the course of 12-month MMT in Vietnamese mountainous provinces.

**Methods:**

We conducted a longitudinal study among patients from 6 MMT clinics in three mountainous provinces of Vietnam. At baseline, we screened 300 participants and after a 12-month treatment, 244 participants remained to involve in the study. World Health Organization Quality of Life short form instrument (WHOQOL-BREF) was used to measure the QoL of patients. The magnitude of the changes was extrapolated.

**Results:**

There were significant changes in all four dimensions of quality of life measured by WHOQoL-BREF between baseline and 12 months with the effect size ranged from 0.21 to 0.24. Increased age, being workers compared to unemployment and having health problems or comorbidities were positively related to reduced scores of QoL.

**Conclusions:**

To conclude, we observed a significant increase in the QoL level regarding physical, psychological, social relationships and environmental aspects among people who used drug after 12 months of MMT. Findings from this study emphasize the role of continuously receiving MMT treatment among people using drugs to enhance their QoL.

## Background

There are an estimated 12–21 million opiate users globally, with Europe and Asia remaining as the two major global markets of consumption and heroin is this region’s most popular form of opiate consumption [1]. People who inject drugs (PWID) were the most-at-risk population for the transmission of Human Immunodeficiency Virus (HIV) via sharing and reusing injection equipment with 22 times higher than the general population [2, 3]. Substantial efforts have been made to expand the harm reduction programs, especially methadone maintenance therapy (MMT) which is used as an alternative for other opioids [4–6].

In recent decades, Vietnam has experienced one of the fastest-growing HIV epidemics in Asia, largely driven by injection drug use and sex work [7]. People who use drug in Vietnam tripled between 1994 and 2004 [8]. Despite this, further action is underway to minimize the chances of both drug use and HIV becoming major endemic problems among these vulnerable groups throughout the country, which considers drug use a destructive “social evil”. Since the introduction of the first pilot MMT clinic in 2008, the Vietnamese government has scaled up the delivery of these programs in nationwide as a core component of the HIV reduction strategy in Vietnam [9]. These programs have been recognized that reduced psychological distress [10], stigma [11, 12], alcohol and tobacco use [13], economic vulnerability [14], risky sexual behaviors [15], and unemployment [16].

Previous studies indicated that MMT patients’ health-related quality of life can be comparable to the general population [17, 18]. These studies only mentioned health aspects, while other perspectives in patients’ life such as social relationships or environment also play crucial roles. As such, measuring quality of life (QOL) of MMT patients should be paid special attention. Higher QoL is closely associated with higher level of MMT adherence and higher likelihood of community reintegration via improving workability [19–21]. However, most of the studies were conducted in urban and rural settings, while a lack of evidence was documented in mountainous areas, where the accessibility of health care services among drug users is constrained [10, 11, 13, 15, 22, 23]. Therefore, this study aimed to assess QOL, mental disorders and their associated factors amongst people using drugs over the course of 12-month MMT in Vietnamese mountainous provinces.

## Methods

### Study design and sample

A pre-post study with no control group was performed from December 2014 to December 2015 in three mountainous provinces including Dien Bien, Lai Chau, and Yen Bai. There were 6 clinics selected in the study, three clinics were Provincial AIDS Center in Dien Bien, Lai Chau, Yen Bai City, and three clinics were District Health Center in Thanh Xuong, Phong Tho and Nghia Lo. These clinics met the following inclusion criteria 1) In Dien Bien, Lai Chau and Yen Bai provinces; 2) Covered both urban and rural regions of mountainous provinces 3) Covered both provincial and district levels of Vietnam health system and 4) Currently offering MMT services based on the guideline of Ministry of Health. To enroll in the study, participants had to meet the following criteria [1] Aged 18 years old or above [2]; Currently receiving MMT services at one of the aforementioned clinics during the time of the study for at least 3 months [3]; Having the physical and psychological capable to answer questions from the interviewers [4]; Provided informed consent to a member of the study team present at one of the study sites.

We used a convenience sampling approach to enroll participants from selected clinics at the provincial and district level. Participants were invited to take part in the study in the first month and 12th-month of the MMT program. At baseline, we screened 300 participants, all of whom met the eligibility criteria for study participation; after a 12-month treatment, 244 participants still remained to involve in the MMT program. We dropped out 56 participants with the retention rate of 81.3% because they withdrew from the treatment program. As in our settings, only male patients enrolled in the treatment program; thus, 100% of patients in our study were males.

### Measures and instruments

A structured questionnaire was used to face-to-face interview patients. Data collectors included experts in substance abuse fields and staff from Hanoi Medical University who had well training in conducting community surveys. The questionnaire was carefully piloted among 10 patients of different socio-economic backgrounds to test the appropriateness of language and logical issues.

This is a study from the project which aimed to access the health status and effectiveness of the MMT program among illicit drug users in the mountainous provinces of Vietnam over the 12-month follow-up. The primary objective of the study was exploring the potential changes in the drug- and HIV-related risk behaviors of MMT users and associated factors [24]. In addition, finding from our study demonstrated the secondary outcomes which focusing on changes in quality of life of those subjects. Socio-economic characteristics of participants including age, ethnicity, educational level, marital status, and occupation were described elsewhere [24]. Overall, the majority of the participants were 30–49 years old (74.2%), Kinh people and lived with spouse/partner (58.0%). There were more than half of the participants attending lower high school (57.0%) and those were freelancer accounted for 45.9%.

#### Risk behaviors

We asked participants of how long their history of drug use was, injecting drugs within the last 30 days, the history of re-use/sharing of needles. We also asked participants about their alcohol practice in the last 12 months and whether they currently smoke tobacco. Sexual behavior was recorded by asking participants if they utilized a condom in the last sexual practice if they had sex with female sex worker in the last 12 months.

#### Health status and quality of life

To capture the self-report health status of participants, they self-reported whether having any health problems (acute symptoms or chronic illnesses) in the previous 30 days of being hospitalized in the last 3 months. We also asked participants to report the comorbidities such as Hepatitis B (HBV), Hepatitis C (HCV), HIV/AIDS and other opportunistic infections. In terms of QoL, self-reported QoL was captured using a version of the WHO Quality of Life short-form instrument (WHOQoL-BREF). This tool was translated into Vietnamese based on the protocol offered by the WHOQOL Group [25]. A forward-backward translation was used to secure the appropriateness of language and logical issues. Any differences were subsequently reviewed and discussed within and between groups. The WHOQoL-BREF has 26 items measuring QOL in four domains: Physical Health, Psychological Health, Social Health, and Environmental Health. Respondents answered each dimension using a five-point Likert scale which ranged from 1 (Not at all) to 5 (Completely). Each domain has a score ranging from 0 to 100, which a higher score indicates higher QoL in each domain. Validation of the questionnaire with Vietnamese MMT patients was made elsewhere and the Cronbach’s alpha of all domains was 0.81 which reflected good internal consistency reliability of the tool [18].

### Statistical analysis

Any differences with a *p*-value of < 0.05 were considered statistically significant. ANOVA, χ2 and Fisher’s exact tests were utilized to determine differences in characteristics between provinces (Dien Bien, Lai Chau, and Yen Bai). To measure the magnitude of the effect of the MMT program, we used the effect size. To estimates the relationship between two binary variables (2 × 2), Cramér’s Phi was performed and using Cramér’s V in case of variables having higher than two levels. Cohen’s d was used to estimate the difference of two population means. In order to interpret the level of classification, the values of 0.01, 0.1, 0.3, and 0.5 were equal to four classifications very small, small, medium and large effect sizes respectively [26]. In this study, we utilized STATA version 14.0 (StataCorp LP, College Station, Texas, USA) to analyze collected data. Multivariate Tobit regression was used to determine potential predictors of each domain of the WHOQoL-BREF. Final models were developed by combining stepwise forward selection strategies, including variables if they had *p*-value < 0.2 for the log-likelihood ratio test.

## Results

The information on demographic characteristics was presented in Table 1. Most of our participants were between the ages of 30–49 (71.7%). More than half of the participants were of Kinh ethnicity (58.7%), followed by Thai (27%). 56.2% of participants did not complete high school and 59% lived with a spouse or partner. The majority of participants were freelancers and only 8% reporting that they were unemployed.

**Table 1:** Socio-economic demographic of participants

**Characteristics**	**Dien Bien**		**Lai Chau**		**Yen Bai**		**Total**		***p*-value**
	**n**	**%**	**n**	**%**	**n**	**%**	**n**	**%**	
**Age group**									
< 30	18	17.8	25	25.0	8	8.1	51	17.0	< 0.01
30–39	43	42.6	36	36.0	29	29.3	108	36.0	
40–49	29	28.7	29	29.0	49	49.5	107	35.7	
≥ 50	11	10.9	10	10.0	13	13.1	34	11.3	
**Ethnicity**									
Kinh	62	61.4	39	39.0	75	75.8	176	58.7	< 0.01
Tay	2	2.0	2	2.0	10	10.1	14	4.7	
Thai	35	34.7	41	41.0	5	5.1	81	27.0	
Others	2	2.0	18	18.0	9	9.1	29	9.7	
**Education**									
< High school	55	54.5	73	73.0	40	40.8	168	56.2	< 0.01
High school	38	37.6	20	20.0	47	48.0	105	35.1	
> High school	8	7.9	7	7.0	11	11.2	26	8.7	
**Marital status**									
Single	31	30.7	28	28.0	20	20.2	79	26.3	0.01
Live with spouse/ partner	56	55.5	66	66.0	55	55.6	177	59.0	
Widow/ Divorced	14	13.9	6	6.0	24	24.2	44	14.7	
**Occupation**									
Unemployment	8	7.9	7	7.0	9	9.1	24	8.0	0.14
Freelancer	83	82.2	88	88.0	78	78.8	249	83.0	
Worker/ White-collar worker	1	1.0	1	1.0	7	7.1	9	3.0	
Others	9	8.9	4	4.0	5	5.1	18	6.0	

Table 2 illustrates the health risk behaviors of participants. About 62.6% of participants reported a history of drug use spanned between 2 and 10 years and 9% had a history of re-using /sharing needles. More than two-thirds of participants still injected drugs within the last 30 days (68.3%). About 62.6% of participants reported a history of drug use spanned between 2 and 10 years and 9% had a history of re-using /sharing needles. While 87.3% of participants reported that they currently smoked, 54% reported using alcohol in the last 12 months. Approximate one-fifth of participants had ever had sexual intercourse with sex-worker (16.5%) and 50.7% of participants used a condom in their last sexual encounter.

**Table 2:** Risk behaviors among participants

**Characteristics**	**Dien Bien**		**Lai Chau**		**Yen Bai**		**Total**		***p*-value**
	**n**	**%**	**n**	**%**	**n**	**%**	**n**	**%**	
**Duration of drug use**									
< 2 years	2	2.0	7	9.6	4	4.1	13	4.8	0.73
2–5 years	28	28.3	21	28.8	30	30.6	79	29.3	
5–10 years	35	35.4	22	30.1	33	33.7	90	33.3	
10–20 years	26	26.3	18	24.7	24	24.5	68	25.2	
> 20 years	8	8.1	5	6.9	7	7.1	20	7.4	
**Drug-injection in the last 30 days**	31	19.0	21	13.5	35	22.2	87	18.2	0.13
**Re-use/sharing of needles**	13	12.9	4	4.0	10	10.1	27	9.0	0.08
**Currently smoking**	91	90.1	92	92.0	79	79.8	262	87.3	0.02
**Alcohol practice in the last 12 months**	66	65.4	58	58.0	39	39.8	163	54.5	< 0.01
**Using condom in the last having sex**	49	49.5	49	51.6	49	51.0	147	50.7	0.96
**Had sex with sex workers**	16	16.2	11	11.6	21	21.7	48	16.5	0.17

The change in health status and QoL of participants were performed in Table 3. Slightly higher participants reported being hospitalized in the last 3 months (2.1 and 5.4%) but the change was not statistically significant. About one-fourth of participants having HCV at the baseline and 12-month follow-up (22.5 and 25.7%). More than 10% of participants had a comorbidity of HIV/AIDS in both baselines and after 12 months of treatment (11.1 and 13.4% respectively). There were significant changes in all four dimensions of quality of life measured by WHOQoL-BREF with the effect size ranged from 0.21 to 0.24.

**Table 3:** Changes in health status and quality of life among participants

**Characteristics**	**Baseline**		**Follow-up**		***p*-value**	**Effect size**
	**n**	**%**	**n**	**%**		
**Being hospitalized in the last 3 months**	5	2.1	13	5.4	0.05	0.09*
**Having any health problem in the last 30 days**	32	13.1	24	10.0	0.28	0.05*
**Comorbidities**						
HBV	22	9.0	29	12.2	0.25	0.05*
HCV	55	22.5	61	25.7	0.41	0.04*
Opportunistic infections	4	1.6	6	2.5	0.50	0.03*
HIV/AIDS	27	11.1	32	13.4	0.44	0.04*
**Quality of life (WHOQoL- BREF)**	**Mean**	**SD**	**Mean**	**SD**	***p-value***	**Effect size**
Physical health	60.1	12.9	63.1	12.4	0.02	0.24^†^
Psychological	61.4	12.4	64.3	11.8	0.01	0.24^†^
Social relationships	50.5	14.8	53.7	15.9	0.01	0.21^†^
Environment	59.1	11.4	61.7	11.2	0.01	0.23^†^

*Cramér’s Phi test; ^†^Cohen’s d test

Factors associated with quality of life of participants were described in Table 4. Participants in the age from 30 to 39 were positively associated with reduced score of psychological (Coef = − 6.41; 95%CI = − 11.03; − 1.79) and environment aspect (Coef = − 4.84; 95%CI = − 9.29; − 0.39) compared to those under 30 years old. In addition, participants who were workers/ white-collar workers (Coef = − 11.59; 95%CI = − 22.13; − 1.05) and being hospitalized in the last 3 months (Coef = − 15.58; 95%CI = − 26.70; − 4.46) had a lower score of psychological dimensions. Those who reported any health problems in the last 30 days were associated with a lower score of both physical health (Coef = − 6.55; 95%CI = − 12.17; − 0.93) and psychological (Coef = − 5.84; 95%CI = − 11.41; − 0.28). Regarding comorbidities, having HIV/AIDS was related to a reduced score of physical health (Coef = − 6.44; 95%CI = − 11.83; − 1.05) while suffering from HBV was associated with a reduced score of environment aspects (Coef = − 7.49; 95%CI = − 13.08; − 1.89).

**Table 4:** Factors associated with quality of life among participants

**Characteristics**	**Physical health**		**Psychological**		**Social relationships**		**Environment**	
	**Coef.**	**95% CI**	**Coef.**	**95% CI**	**Coef.**	**95% CI**	**Coef.**	**95% CI**
**Age groups** (vs < 30)								
30–39			−6.41***	−11.03; −1.79			−4.84**	−9.29; −0.39
40–49			−4.68**	−9.27; −0.08			−4.18*	−8.71; 0.35
**Occupation** (vs Unemployment)								
Freelancer	4.89*	−0.86; 10.64						
Worker/ White-collar worker			−11.59**	−22.13; − 1.05				
Others	11.34**	1.75; 20.94						
**Being hospitalized in the last 3 months** (Yes vs No)			−15.58***	−26.70; −4.46				
**Having any health problem in the last 30 days** (Yes vs No)	−6.55**	−12.17; −0.93	−5.84**	− 11.41; − 0.28				
**Comorbidities** (Yes vs No)								
HBV			−5.25*	−11.00; 0.49			−7.49***	−13.08; − 1.89
HIV/AIDS	−6.44**	− 11.83; − 1.05						

*p < 0.05; **p < 0.01; ***p < 0.001

## Discussion

To our knowledge, this one of the first studies that examine the change of quality of life with regard to before and 12-months after MMT treatment among people living in the mountainous areas in Vietnam. The findings of the study showed a significant increase in quality of life, especially in physical health and psychological aspect after a 12-month MMT program. Moreover, the socio-economic characteristics such as age and occupation, and health status were found to significantly associated with the quality of life of patients following MMT treatment.

Our study reported a mostly similar range of average QoL in each domain when compared to other studies of MMT patients in Asia using the WHOQoL-BREF [27]. In addition, HRQOL in our study was higher than that in other groups/specific populations such as elderly people [28], people with hypertension [29]. This can be explained that elderly people normally have to suffer from health problems in performing their usual activities [30] or non-communicable diseases (NCDs) patients have to undergo lifelong treatment which may cause physical pain, discomfort, and sleep problems [31]. However, our results were higher than the quality of life score regarding four domains among HIV-infected patients in the previous study in Vietnam [32]. Sociocultural differences in the valuation of health states and perceptions/stigmatization of drug use and HIV can make QoL comparisons potentially difficult, even if they have been captured using cross-culturally validated instruments like the WHOQoL-BREF [33]. Moreover, among four domains of quality of life, social relationships obtained the lowest score at the base line and after 12 months follow-up. The lack of social relationships can be explained by the fact that people using illicit drug have to take a long time to regain trust in their family and other relatives [34].

The reliability of the WHOQoL-BREF in this area of study has been demonstrated previously by several longitudinal studies on MMT in Vietnam and other parts of Asia, all of which demonstrate improvements in QoL using the WHOQoL-BREF [18, 35–37]. Findings from our study on the improvement of quality of life after 12-months of the MMT program are critical evidence that demonstrates the effectiveness of MMT in enhancing the health status of illicit drug users. In this study, all subdomains of QoL at 12-months follow-up were significantly higher among people who used drug receiving MMT, which is consistent with previous studies [34, 36, 38]. Besides the prevention of relapsing drug use, continuous MMT also related to the persistent improvement of QoL which may lead to better health outcomes, early reintegration into society, a lower rate of drug-related crimes [38]. However, in comparison with previous studies, the magnitude of the effect in this study was small which may be attributed to different study settings and stages of treatment. In our case, study was conducted in mountainous areas where have different ethnicities and lower educational level compared to urban regions. In addition, patients in our study have already received MMT program for at least 3 months, while other studies assessed patients who were firstly exposure to MMT. The previous study revealed that at the initiation of treatment, the patient often suffered from a crisis situation and entered treatment with poor condition, which may lead to the low QoL at admission [39]. Therefore, a limited study on drug users undergoing MMT in these remoted areas may trigger the lack of attention to the vulnerable population and deteriorate the effectiveness of MMT scale-up.

Our study also found that increased age was associated with lower QoL in psychological and environmental aspects. This result was similar to previous work that has found age to be associated with lower-scoring on the Psychological, Social and Environmental domains [27, 35, 40]. A previous study examining the health status of elderly patients using MMT reported that advanced age was related to more chronic diseases, suboptimal adherence to taking medicine schedules [41]. Older patients, especially in mountainous areas - our context, have to face problems in accessing clinics because of the geographical barriers or in performing usual activities regarding physical health [23]. In addition, we also found that participants who were workers were negatively associated with better QoL scores in the psychological dimension. Among MMT patients in China, education and unemployment were also related to the lower score of psychological health on the WHOQoL-BREF [40]. A study of with Fei et al., (2016) revealed the negative association between being employed and mental illness among MMT patients [27]. However, a previous study among African-American participants showed a contrast result as the MMT program increased the patients’ ability to work and having a job related to a better quality of life [42]. The difference can be explained by the stigma and discrimination for MMT patients, especially in remote areas where people lack knowledge about drug problems in society or have negative opinions about drug users [11]. Many employees who are illicit drug users were denied for job applications or got fired when disclosing their addiction treatment processes [16]. Therefore, the importance of public MMT funding and individual employment should not be discounted as vital to the social reintegration and economic stability of recovering drug users in Vietnam [14, 18, 43].

Being hospitalized in the last 3 months or having health problems in the last 30 days also related to the decrease of QoL regarding both physical and psychological aspects. Heroin users tend to have very stable and lengthy patterns of use that are often characterized by severe consequences and a high degree of heterogenous (neuro) physiological, psychological and socioeconomic morbidity [44, 45]. In addition to the MMT program, patients who have acute or chronic diseases may suffer from the financial burden because of paying for treatment fees and health services [46]. Thus, they might undergo more psychological distress than other people. Having HBV as comorbidities also associated with reduced score of environmental factor. Among other outcomes, the consequences of chronic drug use often manifest as difficulty in obtaining stable housing [47] and employment [44] important factors when considering QoL related to the environment. Moreover, MMT patients who suffer from HIV/AIDS were related to a reduced score of physical health because they may have severe side effects such as gastrointestinal reactions, systemic discomforts or metabolic dysfunctions [48].

The findings of this study are critical reference points for policymakers to enhance the effectiveness of interventions that focus on increasing QoL of MMT patients both on national and regional levels. First, our study further emphasizes the need for scaling-up of MMT programs to increase the level of QoL of MMT patients. More resources should be allocated in mountainous and remote areas as the inherent challenges which act as main barriers in accessing MMT programs. Second, we also suggest the necessity to consider socio-economic differences in designing harm reduction interventions. Obtaining a job plays a vital role for MMT patients to build social reintegration but the stigma and discrimination should be taken into account and eliminated. Thirds, patients suffering from other health problems besides MMT treatment are vulnerable populations; hence more attention should be paid to avoid mental health problems and aggravated conditions of physical health.

The limitations of this study included at first, the limited ability to be generalized of the sample since it was a facility-based survey that recruited naïve patients but only those who accessed MMT clinics. Secondly, the cross-sectional design did not allow establishment of causal pathways of independent factors and QoL outcomes of the study. Nevertheless, we have improved the validity of this assessment by purposively selecting clinics in different regions and settings. Thirdly, we also considered a pre- and post- evaluation with no control group as a weakness of the study design. Another limitation can be acknowledged is that the Type I error rate was inflated due to the multiple tests. Finally, face-to-face interview patient was used which may lead to recall bias.

## Conclusions

To conclude, we observed a significant increase in the QoL level regarding physical, psychological, social relationships and environmental aspects among people using drugs after 12 months of MMT. Findings from this study emphasize the role of continuously receiving MMT treatment among drug users to enhance their QoL. Given the inherent challenges in mountainous areas, it is recommended to policymakers to craft more interventions to support vulnerable subjects such as those who have served health problems besides MMT treatment or under socio-culturally sensitive.

## Data Availability

The datasets used and analyzed during the current study are available from the corresponding author on a reasonable request.

## Abbreviations

AIDS: Acquired Immunodeficiency Syndrome
HBV: Hepatitis B Virus
HCV: Hepatitis C Virus
HIV: Human Immunodeficiency Virus
MMT: Methadone Maintenance Treatment
PWID: People Who Inject Drugs
QoL: Quality of life
WHOQoL-BREF: World Health Organization Quality of Life short form instrument
